# Unravelling a can of worms

**DOI:** 10.7554/eLife.07431

**Published:** 2015-04-16

**Authors:** Alejandro Sánchez Alvarado

**Affiliations:** Howard Hughes Medical Institute, Stowers Institute for Medical Research, Kansas City, United Statesasa@stowers.org

**Keywords:** Platyhelminthes, phylogeny, parasitism, RNA-Seq, other

## Abstract

Understanding the evolutionary relationships between species could help researchers select better model organisms to study in the laboratory.

**Related research article** Laumer CE, Hejnol A, Giribet G. 2015. Nuclear genomic signals of the ‘microturbellarian’ roots of platyhelminth evolutionary innovation. *eLife* **4**:e05503. doi: 10.7554/eLife.05503

**Image** Despite their diversity and abundance, the biology of many flatworms has not previously been studied in depth

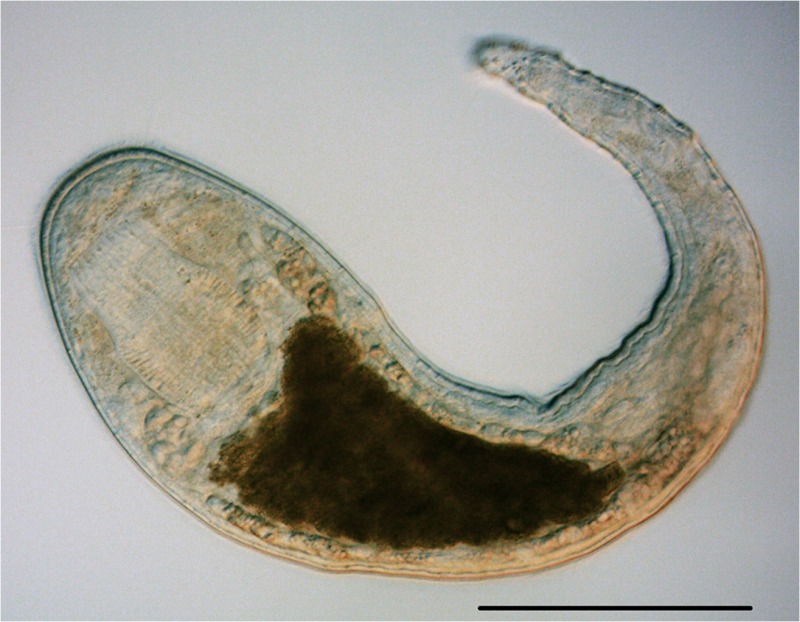
IMAGE CREDIT: CHRISTOPHER E LAUMER

The written accounts and reasoning associated with how the species we currently use for biomedical research were selected as model systems have rarely included weighty consideration of either their evolutionary or natural histories (Alfred and Baldwin, 2015). Instead, organisms such as *Drosophila*, *Caenorhabditis elegans*, zebrafish and mice were primarily chosen as model organisms for purely practical reasons. For example, many model organisms produce large numbers of offspring, have features that make them easy to examine (such as transparent embryos), or are easy to domesticate and look after in the laboratory. Paradoxically, while this approach has been remarkably successful in advancing our understanding of life, it has also made us acutely aware of how much more biology we have yet to comprehend.

Some have argued that further research into the model organisms that dominate much of biomedical research today could fill in the many gaps that exist in our understanding of life, but these organisms give a biased and ultimately poor statistical representation of the ∼30 million species of animals that populate our planet (Brusca and Brusca, 2003). Our best chances of uncovering new biology and acquiring a truly transformative understanding of life are therefore to be found in the laboratory of nature. As technology advances and allows us to examine aspects of biology that were not previously accessible to scientific interrogation, we may want to reconsider how we choose animals as new model systems. Instead of selecting them purely on the biological attributes they conveniently exaggerate for our scientific interests, we could also consider the ecological and evolutionary histories that may have helped produce such attributes.

These considerations are particularly timely in view of current efforts to develop and characterize invertebrate model systems for studying regeneration and parasitism. Now, in *eLife*, Christopher Laumer of Harvard University, Andreas Hejnol of the University of Bergen and Gonzalo Giribet, also from Harvard, have unravelled the phylogenetic tree of the Platyhelminthes, which are more commonly known as the flatworms (Laumer et al., 2015).

Animals possessing bilateral symmetry are presently grouped into three main branches in the metazoan tree of life. The Deuterostomes (the evolutionary lineage to which humans belong) are represented by a number of model organisms including mice, fish, sea squirts, sea urchins and, of course, humans. The second branch, the Ecdysozoa, is presently represented in biomedical research by the fruit fly *Drosophila melanogaster* and the roundworm nematode *C. elegans*. However, the third branch, the Lophotrochozoa, remains among the most undersampled and understudied collection of complex organisms on the planet. This is despite the fact that it encompasses a collection of animals with an assortment of body plans, biological attributes, and ecological adaptations that is unmatched by the other two bilaterian metazoan branches combined. Within the Lophotrochozoa, no group of animals manifests these attributes as clearly as the Platyhelminthes: the diversity of body plans, developmental plasticity and ecological adaptations displayed by these flatworms is remarkable.

Laumer, Hejnol and Giribet report on a comprehensive analysis of the evolutionary relationships among the Platyhelminthes using a survey of genomes and transcriptomes that represents all free-living (i.e., non-parasitic) flatworm orders. This work is the first of its type for the Platyhelminthes and ultimately provides a modern hypothesis that should help us to understand how this extraordinarily diverse group of animals evolved.

By comparing hundreds of nuclear protein coding genes, Laumer et al. were able to derive a phylogeny with at least two important and intriguing attributes. Firstly, key evolutionary transitions within the Platyhelminthes unexpectedly featured the involvement of ‘microturbellarian’ (microflatworm) groups (Figure 1). Secondly, a novel scenario that explains the interrelationships between free-living and parasitic flatworms provides unique opportunities for shedding light on the origins and biological consequences of the parasitic lifestyle in these animals.

**Figure 1.**
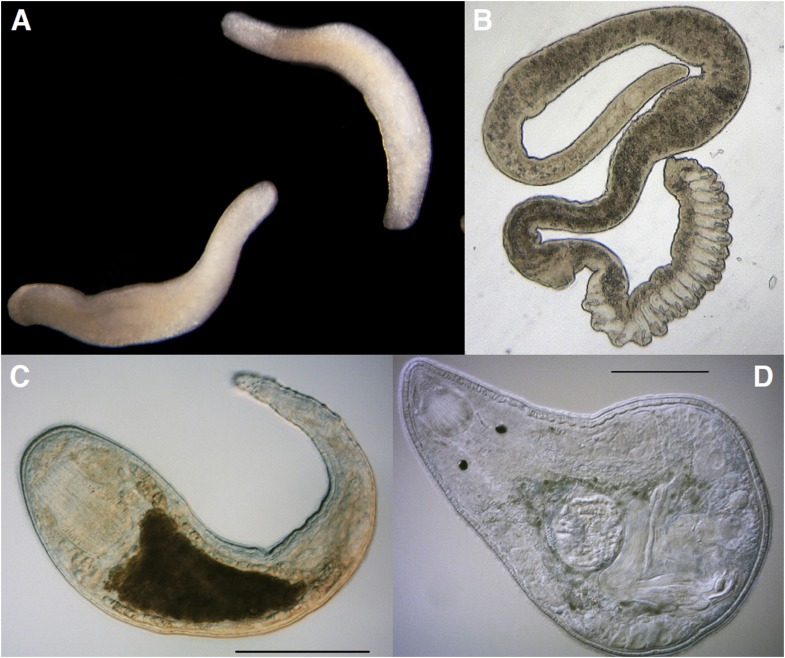
The Platyhelminthes show a diverse range of body plans and developmental characteristics. (**A**) Two adult *Bothrioplana semperi* (Bothrioplanida) swimming in different directions. Adults are approximately 1.5 mm outstretched. Collected from near Saw Mill brook, Estabrook Woods, Concord, MA. (**B**) Adult of an undescribed species of *Polystyliphora* (Proseriata), collected from the interstices of marine sand in Bocas del Toro, Panama. Scale bar unavailable. (**C**) Adult specimen of an undescribed blind species of *Microdalyellia* (Rhabdocoela), collected in fresh water from Mt. Blue Spring, Wompatuck State Park, MA. (**D**) Adult, undetermined species of *Nannorhynchides* (Rhabdocoela), collected in brackish water at low tide near Sage Lot Pond outlet, Mashpee, MA. Scale bars: 100 µm. Samples in panels **A**, **C** and **D** photographed by Christopher E Laumer. Sample in panel **B** photographed by Marco Curini-Galletti.

The Tricladida order of flatworms contains the important model system *Schmidtea mediterranea*, which is used to study tissue regeneration and development. An intriguing point raised by the phylogenetic tree produced by Laumer et al. is that this order may be evolutionarily equidistant to two other orders (Prolecithophora and Fecampiida). This new relationship will have to be taken into account from this point onward when considering how the regenerative properties displayed by these three groups of animals evolved.

The work of Laumer et al. makes it clear that we should embrace an approach that involves morphological studies, evolutionary developmental biology and evolutionary genomics when selecting organisms for experimental interrogation. The evidence reported for the importance of microturbellarians (Figure 1) in the evolution of Platyhelminthes may ultimately prove to be the single most important contribution of the present body of work. Microturbellarians have not captured the attention of researchers like the best-known branches of the clearly much larger and phylogenetically diverse flatworms (e.g., planarians, polyclads, and neodermatans). It is my suspicion that this paper will bring an end to their relative obscurity.
